# lncRNA CDKN2B-AS1 is downregulated in patients with ventricular fibrillation in acute myocardial infarction

**DOI:** 10.1371/journal.pone.0304041

**Published:** 2024-05-21

**Authors:** Ricardo Pan-Lizcano, Lucía Núñez, Pablo Piñón, Guillermo Aldama, Xacobe Flores, Ramón Calviño-Santos, José Manuel Vázquez-Rodríguez, Manuel Hermida-Prieto

**Affiliations:** 1 Instituto de Investigación Biomédica de A Coruña (INIBIC), Grupo de Investigación en Cardiología, Complexo Hospitalario Universitario de A Coruña (CHUAC-SERGAS), GRINCAR-Universidade da Coruña (UDC), A Coruña, Spain; 2 Departamento de Ciencias de la Salud, GRINCAR Research Group, Universidade da Coruña, A Coruña, Spain; 3 Instituto de Investigación Biomédica de A Coruña (INIBIC), Servicio de Cardiología, Complexo Hospitalario Universitario de A Coruña (CHUAC-SERGAS), Universidade da Coruña (UDC), A Coruña, Spain; 4 CIBERCV (Centro de Investigación Biomédica en Red Enfermedades Cardiovasculares), Instituto de Salud Carlos III, Madrid, Spain; Free University of Berlin, GERMANY

## Abstract

Ventricular fibrillation (VF) in acute myocardial infarction (AMI) is the main cause of deaths occurring in the acute phase of an ischemic event. Although it is known that genetics may play an important role in this pathology, the possible role of long non-coding RNAs (lncRNA) has never been studied. Therefore, the aim of this work is to study the expression of 10 lncRNAs in patients with and without VF in AMI. For this purpose, the expression of CDKN2B-AS1, KCNQ1OT1, LIPCAR, MALAT1, MIAT, NEAT1, *SLC16A1-AS1*, *lnc-TK2-4*:*2*, *TNFRSF14-AS1*, and *UCA1* were analyzed. After the analysis and Bonferroni correction, the lncRNA *CDKN2B-AS* showed a statistical significance lower expression (P values of 2.514 x 10^−5^). *In silico* analysis revealed that six proteins could be related to the possible effect of lncRNA *CDKN2B-AS1*: AGO3, PLD4, POU4F1, ZNF26, ZNF326 and ZNF431. These *in silico* proteins predicted to have a low cardiac expression, although there is no literature indicating a potential relationship with VF in AMI. Thus, the lncRNA CDKN2B-AS1 shows a significant lower expression in patients with VF in AMI *vs* patients without VF in AMI. Literature data suggest that the role of CDKN2B1-AS is related to the miR-181a/SIRT1 pathway.

## Introduction

Ventricular fibrillation (VF) in acute myocardial infarction (AMI) is the main cause of deaths occurring in the acute phase of an ischemic event and studies have suggested that the frequency of VF in AMI is 3%-12% of all AMI cases; however, the real number is beyond doubt higher as many are found dead [1,2]. It is important to note that VF in AMI is one of the main challenges to clinicians, because in more than half of the cases, coronary artery disease in these patients has not previously been recognized clinically, and VF in AMI or cardiac sudden death occurs as its first symptom [2,3].

Although the molecular mechanisms involved in VF in AMI are mainly unknown, the interaction between genetic and environmental factors [4,5] has been proposed. The weight of the genetic basis of the disease has been established by the identification of mutations and rare variants associated with VF in AMI [2].

Recently, elements involved in the epigenetic regulation of genes related to cellular remodeling, fibrosis and cardiac conduction that may play a determining role in VF in AMI, have been studied. One of the main epigenetic components studied is the expression of microRNAs (miRNA) in VF in AMI. miRNAs are a class of small endogenous RNA molecules between 19 and 22 nucleotides long, single-stranded, non-coding RNAs that act as post-transcriptional regulators, primarily by inhibiting gene expression [5]. Bostjancic *et al* [6] identified a decreased expression of microRNA 133a/b in cardiac tissue in patients with VF in AMI, which was upheld on days 2–7 post-infarction. Moreover, other studies have related levels of different microRNAs to diverse outcomes after cardiac arrest [7–9]. For all these reasons, microRNAs may help to gain insight into the pathophysiology of VF in AMI, improve the prognosis of these patients and become a pharmacological target for new treatments.

However, other epigenetic components such as long non-coding RNAs (lncRNAs), molecules of 200 nucleotides that are not translated into proteins, have not been studied in patients with VF in AMI. This fact is noteworthy because there are two types of evidence suggesting that they may be involved in VF in AMI:

i.) different lncRNAs have been related with AMI [10]: the first AMI-associated lncRNA, myocardial infarction associated transcript-MIAT, was identified through GWAS in 2006 in the largest study performed to date, in which, 3435 patients with AMI were included [11]. Subsequently, through microarrays [12] or qPCRs, different lncRNAs associated with AMI were identified, among which, the following stand out: CDKN2B-AS1 [13,14], KCNQ1OT1 [13], UCA1 [15,16], LIPCAR [17], MALAT1 [14] and the 3 lncRNAs identified in the study by Zhai *et al* [18]: TNFRSF14-AS1, SLC16A1-AS1, and TCONS_00024701. In recent years, novel lncRNAs have been identified through RNASeq such as NEAT1 [19–21]. Of particular interest is the study by Zhong *et al* [22] describing a different lncRNA profile in patients with ST-segment elevation AMI and patients with non-ST-segment elevation AMI. More recently, Zheng *et al* revealed that circulating exosomal lncRNAs ENST00000556899.1 and CAMTA2-AS1 are raised in patients with AMI [23].

ii.) lncRNAs may play a role in arrhythmias [24]: lncRNAs have been described to participate in the development of atrial fibrillation [25–27] and long QT syndrome [28]. Moreover, lncRNAs can contribute to the control of cardiac impulse conduction by regulating intercellular junctions, as it has been described in different animal models [29].

Therefore, there is evidence that lncRNAs play a role in the development of AMI and also in the genesis of different arrhythmias. However, there is no study in the literature that explores the role of lncRNAs in VF in AMI. So, we focused on the expression of 10 lncRNAs (MIAT, CDKN2B-AS1, MALAT, NEAT1, KCNQ1OT1, UCA1, LIPCAR, TNFRSF14-AS1, SLC16A1-AS1,lnc-TK2-4:2) in patients with AMI and VF and their differential expression with respect to patients with AMI without VF. lncRNAs involvement in the physiopathology of the disease, may conduct to the proposal of new therapeutic targets for the treatment of VF in AMI.

## Materials and methods

### Patients and sample

Serum and plasma samples were obtained from patients diagnosed with AMI with elevation of the ST-segment (STEMI) with and without VF. All of them were treated with a primary percutaneous coronary intervention (PPCI) in A Coruña University Hospital (Spain) between January 2019 to January 2023 (recruitment period). Written informed consent was obtained from every patient included in the study. The protocol of this study was in accordance with the principles of the Declaration of Helsinki, and the Galician Research Ethics Committee (ref: 2016/299) had approved it. Blood samples were collected at the time of the hemodynamic procedure and on the same day the centrifugation to obtain serum was performed. Only samples with none signs of hemolysis (colorimetric measure) were stored at -80°C until final analysis. All the samples were included in the collection C.0002483, 2013/109 of National Biobank Network of “Instituto de Salud Carlos III”.

### lncRNA selection

Based on literature search, 10 lncRNA were selected to analyze their association with VF during AMI: *CDKN2B-AS1* [13,14], *KCNQ1OT1* [13], *LIPCAR* [13,17], *MALAT1* [14], *MIAT* [30], *NEAT1* [19–21], *SLC16A1-AS1* [18], *lnc-TK2-4*:*2* [18], *TNFRSF14-AS1* [18], and *UCA1* [15,16].

### Rt-qPCR and expression analysis

Plasma and serum samples were processed with miRNA Serum/Plasma Advanced kit (Qiagen), the same day, reverse transcription reaction [31] was performed using the StaRT Reverse Transcription Kit (Anygenes, Paris, France). After all cDNA samples were obtained a preamplification reaction was done with Specific PreAmplification SpeAmp kit (Anygenes, Paris, France). The equipment used for the qPCR reactions was a Lightcycler 480 Real-Time PCR system (Roche, Basel, Switzerland) using Perfect Master Mix Syber Gr kit (Anygenes, Paris, France). Expression levels were normalized using the gene *RPLP0* as reference, and a Ct value of 35 was chosen as cut-off for expression. To calculate the levels of expression and statistical analysis the program qBase (Biogazelle, Gent, Belgium) was used.

### lncRNA interaction analysis

After the differential expression of the lncRNA molecules in the different groups was evaluated, potential interactions with other molecules were assessed using specialized databases.

The databases selected for the search were LncRRIsearch [32] and LncExpDB [33]. Making use of the name of the statistically significant differentially expressed lncRNA molecules as the main search term, a list of possible interactions was obtained. Results from each search were crossed and the interactions which were in both results tables were analyzed.

### Statistical analysis

The analysis for the variables: sex, dyslipidemia, hypertension, diabetes, and tobacco, were calculated performing a chi-squared test; for the age and body mass index (BMI) the T-test was used.

Statistical analysis of the different expression levels was calculated by qBase (Biogazelle, Gent, Belgium). Data normality was assessed using D’Angostino & Pearson test and, depending on their distribution, either unpaired t-test or Mann-Whitney were performed. As the nature of this study is the comparison of expression levels of different lncRNA, a statistical correction of the P value was needed. Bonferroni correction was performed. As 10 lncRNA were evaluated in this study, the corrected α value was 0.005.

## Results

### Population characteristics

A total of 60 samples were included, 30 patients with AMI with elevation of the ST-segment (Control) and 30 patients with AMI with elevation of the ST-segment and VF. A description of the demographic and clinical data of the population is summarized in Table 1. There were not significant differences between the groups in any of these variables.

**Table 1:** Demographic and clinical data of the studied population.

		With VF (n = 30)	Without VF (n = 30)
**Age (years)**		57.7±10.6	60.9±11.6
**Sex (Masculine, %)**		80.0	76.6
**BMI (kg/m** ^**2**^ **)**		27.6±3.4	27.7±4.1
**Dyslipidemia (%)**		53.3	46.6
**Hypertension (%)**		33.3	40.0
**Diabetes (%)**		13.3	13.3
**Tobacco (%)**		73.3	70.0
**AMI Localization (%)**	**Anterior**	56.7	36.7
	**Septal**	0	3.3
	**Inferior**	33.3	53.4
	**Posterior**	3.3	3.3
	**Lateral**	6.7	3.3
**Vessels affected (%)**	**1**	53.3	43.3
	**2**	16.7	16.7
	**3**	30.0	40.0
**Time of Ischemia (%)**	**<120 min**	20.0	16.6
	**120–360 min**	70.0	43.4
	**>360 min**	10.0	40.0

### lncRNA expression levels

**Fig 1:**
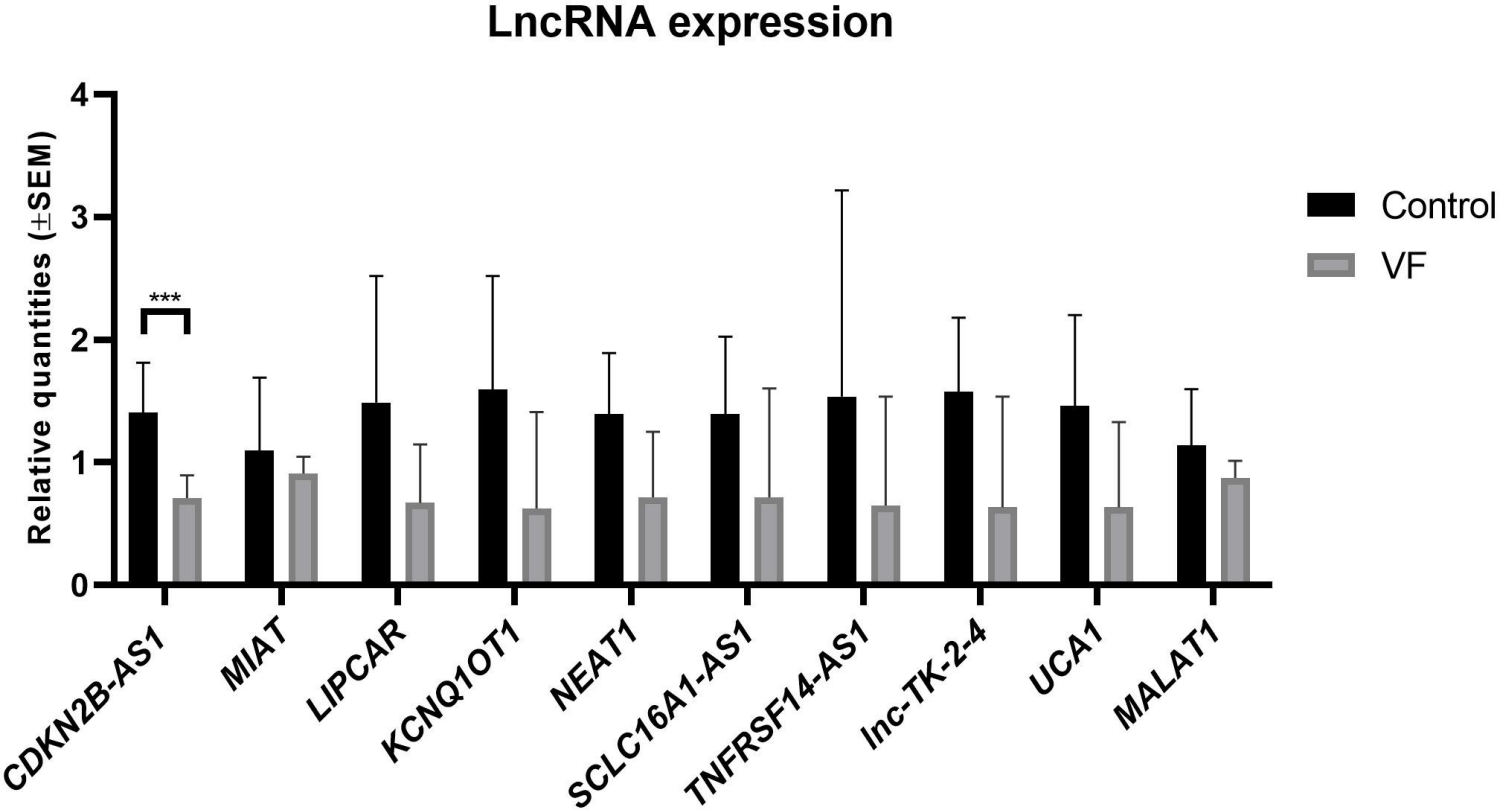
Mean expression level of the 10 studied lncRNA. ***: P value under 0.005.

The data obtained showed that all the lncRNAs studied had a slighter lower mean expression in the VF group compared to Control group (Fig 1). However, the only lncRNA with a statistical significance lower expression, after Bonferroni correction, was *CDKN2B-AS1* (P values of 2.514 x 10^−5^) (Fig 1 and Table 2).

**Table 2:** lncRNA expression in AMI patients with and without VF.

Target	Groups	Mean	95% CI Low	95% CI High	Normal distribution	Test	P value
CDKN2B-AS1	Control	1.407	1.092	1.813	No (P value <0.0001)	Mann-Whitney	2.514 x 10^−5^
	VF	0.711	0.564	0.896			
MIAT	Control	1.099	0.714	1.692	No (P value <0.0001)	Mann-Whitney	7.123 x 10^−3^
	VF	0.910	0.791	1.048			
LIPCAR	Control	1.488	0.879	2.519	Yes (P value 0.189)	Unpaired t-test	3.440 x 10^−2^
	VF	0.672	0.393	1.148			
KCNQ1OT1	Control	1.594	1.176	2.519	No (P value <0.0001)	Mann-Whitney	4.925 x 10^−2^
	VF	0.627	0.279	1.412			
NEAT1	Control	1.395	1.029	1.892	No (P value <0.0001)	Mann-Whitney	0.056
	VF	0.716	0.412	1.249			
SLC16A1-AS1	Control	1.396	0.961	2.027	No (P value <0.0001)	Mann-Whitney	0.0126
	VF	0.717	0.320	1.606			
TNFRSF14-AS1	Control	1.537	0.734	3.218	No (P value <0.0001)	Mann-Whitney	0.198
	VF	0.651	0.275	1.539			
lnc-TK2-4:2	Control	1.578	1.143	2.179	No (P value <0.0001)	Mann-Whitney	0.249
	VF	0.634	0.244	1.645			
UCA1	Control	1.461	0.970	2.201	No (P value <0.0001)	Mann-Whitney	0.280
	VF	0.684	0.353	1.328			
MALAT1	Control	1.142	0.817	1.597	No (P value <0.0001)	Mann-Whitney	0.387
	VF	0.875	0.756	1.013			

### lncRNA interaction

To assess the potential interactions of the CDKN2B-AS1 with different genes, a search of *CDKN2B-AS1* in two databases (LncRRIsearch [32] and LncExpDB [33]), was performed.

These databases differ in the premise used to highlight the interactions. LncRRIsearch uses the minimum energy cost that two molecules would need to interact, stating the lower the energy the most likely are two molecules to interact. Using this database, 100 molecules were shown and annotated (using the CDKN2B-AS1 transcripts ENSG00000240498 and ENST00000428597).

As for LncExpDB, the search was performed using the LncRNA symbol: CDKN2B-AS1, LINC01239. From this database, around 800 molecules were brought up, these molecules were reported as interactions in the literature or other databases. Crossing these two sets of results, six genes were shown to be depicted in both searches (Table 3).

**Table 3:** Molecules that are predicted to interact with CDKN2B-AS1 (results of the crossing search in LncRRIsearch and LncExpDB databases).

Sum of Energy	Min of Energy	Transcript ID	Transcript Name	Gene	Description	Location	ID
-690.68	-30.63	ENST00000392593	PLD4-001	*PLD4*	Phospholipase D family member 4	chr9:21994130-22876986(+)	ENSG00000166428.13
-543.22	-64.1	ENST00000373191	AGO3-003	*AGO3*	Argonaute RISC catalytic component 3	chr9:21994130-22876986(+)	ENSG00000126070.20
-444.47	-30.63	ENST00000370447	ZNF326-004	*ZNF326*	Zinc finger protein 326	chr9:21994130-22876986(+)	ENSG00000162664.17
-403.36	-58.28	ENST00000311048	ZNF431-001	*ZNF431*	Zinc finger protein 431	chr9:21994130-22876986(+)	ENSG00000196705.8
-392.16	-58.93	ENST00000328654	ZNF26-001	*ZNF26*	Zinc finger protein 26	chr9:21994130-22876986(+)	ENSG00000198393.8
-392.12	-31.88	ENST00000377208	POU4F1-001	*POU4F1*	POU class 4 homeobox 1	chr9:21994130-22876986(+)	ENSG00000152192.8

## Discussion

In this study, 10 lncRNA has been analyzed in order to assess their differential expression in patients with or without VF in AMI. CDKN2B-AS1 showed a significant lower expression in patients with VF in AMI *vs* patients without VF in AMI. After the evaluation of the candidate genes that are predicted to interact with CDKN2B-AS1, six genes have been identified: AGO3, PLD4, POU4F1, ZNF26, ZNF326, and ZNF431.

CDKN2B-AS1, also known as ANRIL, is a 3.8 kb length lncRNA located in chromosome 9p21. CDKN2B-AS1 plays a vital role in cell proliferation, aging, inflammation, apoptosis and also as a tumor suppressor [34,35]. In the field of cardiovascular disease, CDKN2B-AS1 has been associated with atherosclerotic vascular disease, coronary artery disease, stroke, aortic aneurysm, and myocardial infarction [36]. Vausort *et al* [13] showed that patients with ST-segment-elevation AMI had significant lower levels of CDKN2B-AS1 than patients with NSTEMI (P<0.001). It is important to note that our data were obtained from a sub-group of ST-segment elevation patients, the ones who present VF during AMI, and that those patients present even lower levels of CDKN2B-AS1 than AMI patients without VF.

In an attempt to search molecules with which CDKN2B-AS1 can interact, a crossing search in LncRRIsearch and LncExpDB databases and six candidates’ genes have been identified: AGO3, PLD4, POU4F1, ZNF26, ZNF326, and ZNF431. AGO3, PLD4, and POU4F1 encoded to the third component of the argonaute RISC catalytic protein, the member 4 of the Phospholipase D Family, and a member of the POU-IV class of neural transcription factors, respectively. These three proteins show a very low expression in the heart [37] and no data associated these genes to cardiovascular diseases. Regarding to Zinc-finger proteins (ZNFs), ZNF26, ZNF326, and ZNF431, they present a moderate expression in heart tissue [37]. It is important to note that ZNFs are one of the most abundant groups of proteins and have a wide range of molecular functions. They are able to interact with DNA, RNA, PAR (poly-ADP-ribose) and other proteins and are involved in the regulation of several cellular processes as transcriptional regulation, ubiquitin-mediated protein degradation, signal transduction, actin targeting, DNA repair, and cell migration [38]. In the field of cardiovascular diseases ZNFs, as GATA factors, are involved in the pathogenesis of congenital heart diseases (CHDs) [39,40]. However, none of the ZNFs that could bind CDKN2B-AS1 have been associated to cardiovascular diseases.

As we have found no target protein in the *in silico* approach, we reviewed the literature in order to understand the potential mechanism of CDKN2B-AS1 in patients with VF in AMI. The role of CDKN2B-AS1 in AMI has not been clearly stablished but it has been proposed that CDKN2B-AS1 is mostly expressed by lymphocytes after MI because it is expressed both by lymphocytes and by monocytes in healthy donors and it is positively associated with lymphocyte count in patients with MI. This observation is intriguing and, together with the concept that lymphocytes play a critical role after MI suggests that ANRIL might be involved in the response of the heart to ischemic injury [13,41,42]. Moreover, *in vitro* experiments used to mimic myocardial ischemia and reperfusion injury related ANRIL downregulation with miR-181a/SIRT1 regulation [43]. It is important to note that Sirtuin 1, encoded by SIRT1, regulates cardiac electrical activity by deacetylating the cardiac sodium channel [44]. Thus, a dysregulation of cardiac electrical activity due to miR-181a/SIRT1 may be a possible explanation for the presence of VF during AMI in patients with lower CDKN2B-AS1.

### Limitations

A significant limitation of the study lies in the sample size. However, it is essential to note that obtaining a significant number of samples is a challenge in this context, as VF occurs in less than 3%-12% of MI cases [1,2]. Another limitation is that there is not a validation cohort, and the reason is, again, the low number of samples due to the low incidence of the disease. More studies will be required to guarantee the generalizability of the findings.

## Conclusions

The lncRNA CDKN2B-AS1 present a significant lower expression in patients with VF in AMI *vs* patients without VF in AMI.

## Supporting information

S1 Checklist (DOCX)

## Abbreviations

AMI: Acute myocardial infarction
STEMI: AMI with elevation of the ST-segment
lncRNAs: Long non-coding RNAs
PPCI: Primary percutaneous coronary intervention
VF: Ventricular fibrillation
